# Leptin into the rostral ventral lateral medulla (RVLM) augments renal sympathetic nerve activity and blood pressure

**DOI:** 10.3389/fnins.2014.00232

**Published:** 2014-08-08

**Authors:** Maria J. Barnes, David H. McDougal

**Affiliations:** ^1^Nutrition and Neural Signaling Laboratory, Pennington Biomedical Research CenterBaton Rouge, LA, USA; ^2^Neurobiology of Metabolic Dysfunction Laboratory, Pennington Biomedical Research CenterBaton Rouge, LA, USA

**Keywords:** leptin, renal sympathetic nerve activity, blood pressure, rostral ventral lateral medulla, neurons

## Abstract

Leptin is a hormone released from adipose tissue. While this hormone normally acts to reduce feeding behavior and increase energy expenditure, in obesity, resistance to these effects occurs even though the hormone is released in large amounts. Although leptin no longer works to suppress feeding in the obese, leptin retains its potent effects on other autonomic functions such as blood pressure regulation. Leptin has been associated with hypertension and increased sympathetic autonomic activity. Therefore, leptin is emerging as a major contributor to the hypertensive state observed in obesity. Sympathetic control of blood pressure is maintained principally by autonomic reflex control circuits in the caudal brainstem. The rostral ventral-lateral medulla (RVLM) is the primary regulator of the sympathetic nervous system, sending excitatory fibers to sympathetic preganglionic neurons to regulate sympathetic control over resistance vessels and blood pressure. Previous studies from our laboratory have shown that neurons in the ventral lateral medulla express leptin receptors (ObRb). Our present study using pseudo-rabies multi-synaptic retrograde tract tracing and immunohistochemical methods revealed that neurons within the RVLM that send sympathetic projections to the kidney express leptin receptors. Acute microinjection of leptin (1 and 3 μg; 40 nL) into the RVLM evoked a significant increase in Mean Arterial Pressure (MAP) and renal sympathetic nerve activity (RSNA). When the 3 μg dose of leptin was preceded with a leptin antagonist, (SLAN-4; 1 ng), it attenuated the cardiovascular response of leptin. Taken together, these data suggest that leptin's actions within the RVLM may influence blood pressure and renal sympathetic nerve activity.

## Introduction

Leptin is an adipocyte-derived hormone which signals the availability of peripheral energy stores. Circulating leptin levels act as a long term signal of the amount of fat stored in white adipose tissue, while short term fluctuations in leptin levels convey information regarding acute changes in caloric intake. This information is integrated centrally by the autonomic nervous system to regulate a variety of homeostatic functions, most notably food intake and energy expenditure (Morris and Rui, 2009; Myers et al., 2009; Galic et al., 2010; Kelesidis et al., 2010). One mechanism by which leptin affects energy expenditure is by increasing sympathetic tone (Eikelis et al., 2003; Morris and Rui, 2009; Myers et al., 2009).

This increase of sympathetic outflow can produce profound effects on various homeostatic functions including the modulation of cardiovascular dynamics such as arterial blood pressure (Friedman, 2002; Correia and Rahmouni, 2006). Leptin also regulates blood pressure via augmentation of renal sympathetic nerve activity (RSNA); events which are believed to play a significant role in the development of hypertension (Hall et al., 2010). This change in RSNA and blood pressure after leptin administration is absent in experimental animals that have defective leptin receptors (i.e., Zucker Rats and db/db mice), suggesting that these effects are leptin receptor mediated (Haynes et al., 1997; Rahmouni et al., 2003).

The majority of leptin signaling studies have been conducted in the hypothalamus, and acute administration of leptin into regions of the hypothalamus associated with control of cardiovascular functions has been shown to increase RSNA and blood pressure (Marsh et al., 2003; Shih et al., 2003; Rahmouni and Morgan, 2007). However, the long form of the leptin receptor (ObRb) is located throughout the central nervous system (Patterson et al., 2011), and there is growing evidence that extra-hypothalamic leptin signaling plays a critical role in autonomic regulation (Myers et al., 2009). In fact, the caudal hindbrain, which contains several populations of preautonomic neurons, may be a critical site for mediating leptin's effect on sympathetic outflow (Grill and Kaplan, 2002; Grill, 2010). For example, acute injection of leptin into the nucleus of the solitary tract (NST), located in the dorsal hindbrain, has been shown to increase RSNA (Mark et al., 2009; Ciriello and Moreau, 2013).

The rostral ventral lateral medulla (RVLM), located within the hindbrain, contains neurons which play a key role in determining peripheral sympathetic vasomotor tone and blood pressure (Guyenet, 2006). The RVLM integrates multiple descending and cervical-thoracic (barosensor) inputs regulating sympathetic outflow. The RVLM is a “pre-sympathetic” structure in that it sends axons to the intermediolateral cell column; the source of sympathetic preganglionic neurons. Our laboratory reported that leptin receptors are expressed by adrenergic/noradrenergic C1/A1 cells located in the ventrolateral medulla (Barnes et al., 2010), which overlaps the RVLM. Therefore, the possibility exists that preautonomic blood pressure neurons in the RVLM may express leptin receptors and that leptin may regulate blood pressure and RSNA directly by increasing the activity of these neurons. The present study was conducted to determine if RVLM neurons projecting through multi-synaptic pathways to the kidney indeed express leptin receptors and whether acute administration of leptin into the RVLM influences RSNA as well as cardiovascular dynamics.

## Materials and methods

### Animals

Male Long Evan rats (8–10 weeks old) obtained from Charles Rivers were used in these studies. All animals were maintained in a room with a 12:12 light-dark cycle with constant temperature and humidity, and given food and water *ad libitum.* All experimental protocols were performed according to the guidelines set forth by the National Institutes of Health and were approved by the Institutional Animal Care and Use Committees at the Pennington Biomedical Research Center.

### Pseudo rabies virus injections

#### Co-localization of ObRb on RVLM neurons with projections to the kidney

Long Evans rats (*n* = 5) were anesthetized with a ketamine (90 mg/kg) and xylazine (9 mg/kg) cocktail. Using aseptic technique, a flank incision was made to expose the left kidney. Animals received two injections (2 μl each) of pseudo rabies virus 152 (PRV), green fluorescent trans-neuronal tracer virus, into the cortex of the kidney using a Hamilton syringe. The injection site was immediately sealed with liquid bandage (Thermo Fisher Scientific, Pittsburgh PA). The kidney was returned into its appropriate position; overlying skin was sutured with Vicryl and the animal was returned to its home cage for recovery. Four (4) days after the injection, animals were anesthetized with urethane (1 mg/kg) and transcardially perfused with 0.1 M Phosphate-Buffered Saline (PBS) followed by 4% paraformaldehyde. Brains were removed and processed for immunohistochemical demonstration of trans-neuronal tract-tracing and leptin receptor expression.

### Immunohistochemistry

The hindbrain was cut into 30 micron thick sections on a freezing microtome, washed three times with 0.1 M PBS, placed in a blocking solution of 10% goat serum (Jackson ImmunoResearch, West Grove PA) containing 0.3% Triton X-100 (Sigma Aldrich, Saint Louis, MO) for 60 min and incubated for 72 h at 4°C in the primary antibody, chicken anti-ObRb [(1:50) (Neuromics, Edina MN)]. Sections were then washed three times with 0.1 M PBS followed by 60 min incubation in Alexa 594 goat anti-chicken antibody [(1:100) (Invitrogen, Grand Island NY)] followed by three rinses with 0.1 M PBS prior to being mounted on slides with ProLong Gold anti-fade reagent (Invitrogen, Grand Island NY). No additional immunohistochemical processing was necessary to visualize the green fluorescent protein expression induced by our PRV injections. Note that heat-induced antigen retrieval method used in our previous studies of leptin receptor expression in the hindbrain (Barnes et al., 2010) led to quenching of the green fluorescent signal induced via our renal injections of PRV. Therefore, comparable ObRb staining was accomplished by both increasing the concentration of the primary antibody to 1:50 and increasing the incubation time from 12 to 72 h, as described above.

### Quantification of immunohistory

Histological sections containing the majority of the anterior to posterior extent of the hindbrain [(9–14.5 mm post Bregma); (Paxinos and Watson, 2007)] were examined for evidence of PRV and ObRb positive cell bodies in the ventral half of the hindbrain. Sections were visualized with an Axioplan 2 upright microscope (Carl Zeiss Microscopy, Thornwood, NY) equipped with a Lambda LS 175W Xenon arc lamp. A FITC filter set (EX HQ487/25, EM GQ535/40, D Q505lp) was used to visualize the green PRV staining, while a CY3 filter set (EX HQ535/50, EM HQ610/75, D Q565lp) was used to visualize the ObRb staining. Images of positive staining were captured using a CoolSnap HQ CCD camera (Photometrics, Tucson, AZ). Slidebook Software (v2.0; Intelligent Imaging Innovations, Denver CO) was used to generate two dimensional montages of the entire ventral hindbrain of each section which demonstrated positive staining using a Plan Apochromat 20×/0.75 NA objective (Carl Zeiss Microscopy, Thornwood, NY). The numbers of PRV and ObRb positive cells within hindbrain nuclei, as well as the numbers of double labeled cells in each image were quantified.

### Physiological effects of leptin injection into RVLM

#### Measurement of renal sympathetic nerve activity and blood pressure

Male Long Evan rats (*n* = 6 per group) were anesthetized with long acting thiobutabarbital (inactin) [(150 mg/kg); (Sigma Aldrich, St. Louis, MO)] which has minimal interference with autonomic reflexes (Buelke-Sam et al., 1978). Using aseptic techniques, a trachea tube was inserted to allow the airway of the animal to remain patent. The left femoral artery was catheterized with PE 20 tubing attached to an AD Instrument transducer for measurement of blood pressure with a PowerLab data acquisition system (AD Instruments, Colorado Springs, CO). The animals were placed in a stereotaxic frame; the occipital plate was removed to expose the hindbrain. A flank incision was made to expose the right kidney and the renal nerve. The renal nerve was separated from the renal vein and renal artery, placed on bipolar platinum-iridium electrodes (A-M Systems, Carlsborg WA) and secured with kwik cast gel (World Precision Instruments, Sarasota, FL). Renal sympathetic nerve activity (RSNA) and mean arterial pressure (MAP) and heart rate (HR) were monitored continuously. Once preparatory surgery and instrumentation was completed, animals were allowed to stabilize for 60 min. A triple barrel glass micropipette (total tip diameter was 150 micron) containing glutamate [(10 mM); (Sigma Aldrich, St. Louis, MO)], saline (0.9%), leptin [(0.3, 1, or 3 μg); (Peprotech, Rocky Hill, NJ)] or a superactive rat leptin antagonist [(1 ng); (SLAN-4); (Shpilman et al., 2011; Gertler and Elinav, 2014); (Protein Laboratories Rehovot)] was lowered into the RVLM at the following coordinates relative to the calamus scriptorius (2.9 mm rostral, 1.9 mm lateral, 2.7 mm ventral). Confirmation of pipette location was accomplished with nano-injections of glutamate (40 nL, 10 mM), as described by Goodchild et al. (1982), which elicited rapid increases in blood pressure. Similar techniques have been used in previous investigations of the RVLM in regulation of autonomic function (e.g., Adams et al., 2007). After confirmation of the injection site via glutamate, animals were allowed to recover for 30 min prior to the start of the experiment. Each animal served as its own control.

Triple barrel pipettes were filled and administered (40 nL) accordingly: glutamate (10 mM)—0.9% saline—leptin (0.3 μg); glutamate—saline—leptin (1 μg); glutamate—saline—leptin (3 μg); glutamate—saline—SLAN-4 (1 ng); glutamate—SLAN-4—leptin (3 μg); glutamate—saline—Chicago Blue.

### Statistics

#### Physiological experiments

Each animal served as its own control. MAP and RSNA were monitored continuously throughout the experiment; measurement of these parameters was analyzed at 4 min intervals. Time course response of MAP is shown in **Figures 4A**, **5A** as a percent change from baseline. Statistical analysis of mean MAPs before and after the first CNS injection were not significantly different (data not shown); therefore, “baseline” mean MAP for each animal was defined as that value at time −15 min. Changes in MAP and RSNA occurring after the second CNS injection were statistically analyzed across all groups.

Raw RSNA was corrected by subtraction of background noise as determined at the termination of the experiment. These corrected values were used to determine the percent change of RSNA from baseline (i.e., mean corrected values obtained 15 min following the first injection). Analysis of the peak MAP responses and RSNA were made using One-Way ANOVA followed by Bonferroni *post-hoc* multiple comparison tests. Statistics were performed using GraphPad Prism Version 5.01 (LaJolla CA). All values are expressed as mean ± s.e.m. *p*-value < 0.05 was considered statistically significant.

## Results

### Histology

#### ObRb positive cells in the ventral medulla were found in the C1/A1 cell group and the ventromedial region

**Figure 1:**
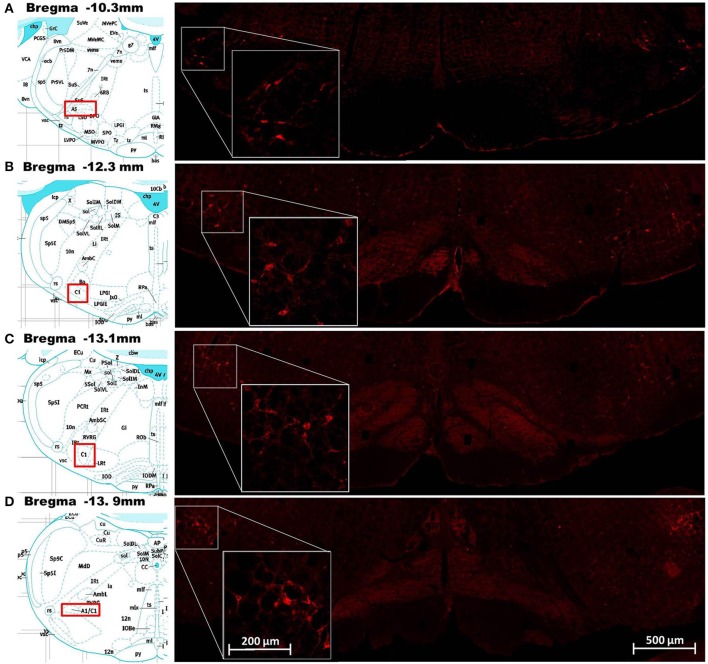
**Demonstration of leptin receptor (ObRb) positive cells at various points along the rostrocaudal axis of the ventral hindbrain**. Images in the right column show corresponding histological staining for ObRb (red labeled cells) in the ventral hindbrain at the level of the A5 cell group **(A)**, RVLM **(B)**, C1 cell group **(C)**, and A1 cell group **(D)**. Insets on the right column show histological staining of ObRb at higher magnification in order to show cellular detail. The A5, RVLM, and C1/A1 cell groups described by Paxinos and Watson (2007) are represented in the pictographs, reprinted with permission, in the left column. Scale bars = 200 microns (insets) and 500 microns.

**Figure 2:**
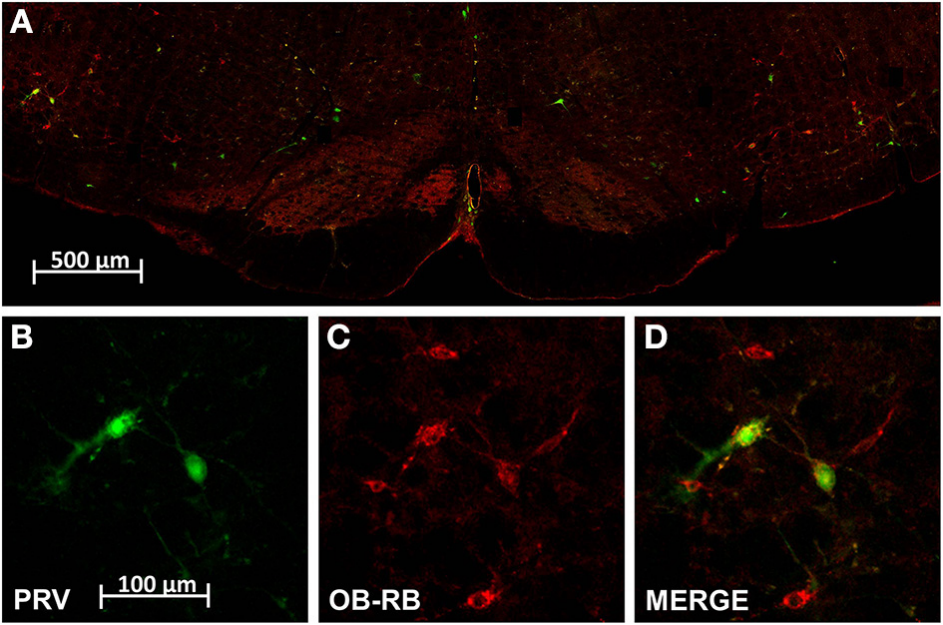
**Dual localization of leptin receptors (ObRb; red) and pseudo rabies virus infected cells (PRV; green) resulting from renal PRV injection. (A)** Is a low power montage of the complete ventral half of the medulla at the level of the RVLM. **(B–D)** Are high magnification of cell cluster in left RVLM shown in **(A)** demonstrating PRV positive RVLM neurons **(B)**, ObRb expressing RVLM neurons **(C)**, and double labeled RVLM neurons **(D)**. Scale bar = 100 microns.

Cells in the ventral hindbrain that were positive for ObRb staining (i.e., expressed leptin receptors) were localized in one of four regions: the C1/A1 cell column, ventromedial medulla (VMM), caudal raphe, and A5 cell group. The ObRb staining in the C1/A1, caudal raphe, and A5 was quite distinct and easily assigned to these medullary nuclei based on Paxinos and Watson (2007) (Figure 1). In contrast, the staining in the VMM was diffuse and often overlapped adjacent nuclei such as the paragigantocellular nuclear subdivisions, the reticular nucleus subdivisions, and the inferior olive (Figure 2A). ObRb positive cells (ObRb^+^) were defined as those cells which displayed extensive cytoplasmic ObRb staining (see insets of Figures 1, 2C), as opposed to cells which merely displayed isolated ObRb punctate staining. An average of 420 ± 87 ObRb^+^ cells were observed in C1/A1, 146 ± 32 ObRb^+^ cells were observed in VMM, 107 ± 87 ObRb^+^ cells were observed in A5, and 26 ± 14 ObRb^+^ cells were observed in the caudal raphe (Table 1).

**Table 1:** **Distribution of leptin receptor (ObRb) expressing and psuedorabies virus (PRV) infected cells in the ventral hindbrain (mean ± s.e.m., *n* = 5)**.

**Hindbrain region**	**Number of ObRb positive cells**	**Number of PRV infected cells**	**Number of double labeled cells**	**% of PRV cells positive for ObRb**
A5	107 ± 11	35 ± 21	30 ± 19	85 ± 4
C1/A1	420 ± 87	88 ± 30	59 ± 19	67 ± 2
VMM	146 ± 32	113 ± 81	50 ± 38	45 ± 7
Caudal raphe	26 ± 14	73 ± 42	16 ± 12	22 ± 13

#### Pseudo rabies (PRV) positive neurons identified in the ventral hindbrain after injection in the cortex of the left kidney

Our PRV kidney injection paradigm labeled cells in C1/A1, VMM, caudal raphe and A5 (Figure 2). An average of 88 ± 30 PRV^+^ cells were observed in C1/A1, 113 ± 81 PRV^+^ cells were observed in VMM, 35 ± 21 PRV^+^ cells were observed in A5, and 73 ± 42 PRV^+^ cells were observed in the caudal raphe (Table 1). PRV^+^ cells in C1/A1 were concentrated in the rostral portion of the cell column, consistent with specific labeling of RVLM neurons. There were no differences in the number of PRV^+^ cell on the contralateral and ipsilateral hemispheres relative to the site of renal injection. These results confirm a number of previous studies which employed PRV injection into the renal cortex (Schramm et al., 1993; Huang and Weiss, 1999; Sly et al., 1999; Weiss et al., 2001), as well as studies employing PRV injected into organs which receive substantial sympathetic inputs (Strack et al., 1989a,b; Sved et al., 2001).

#### A subset of hindbrain neurons projecting through multi-synaptic pathways to the kidney expressed ObRb

A subset of PRV labeled neurons within the hindbrain were also leptin receptor expressing cells (Figure 2). An average of 59 ± 19 double labeled cells were observed in C1/A1, 50 ± 38 double labeled cells were observed in VMM, 30 ± 19 double labeled cells were observed in A5, and 16 ± 12 double labeled cells were observed in the caudal raphe. Thus, approximately 67% of C1/A1 cells that were PRV+ also expressed leptin receptors compared to 45% in VMM, 22% in caudel raphe and 85% of all PVR^+^ cells in A5 (Table 1).

### Physiology

#### RVLM identified by nano-injection of glutamate

**Figure 3:**
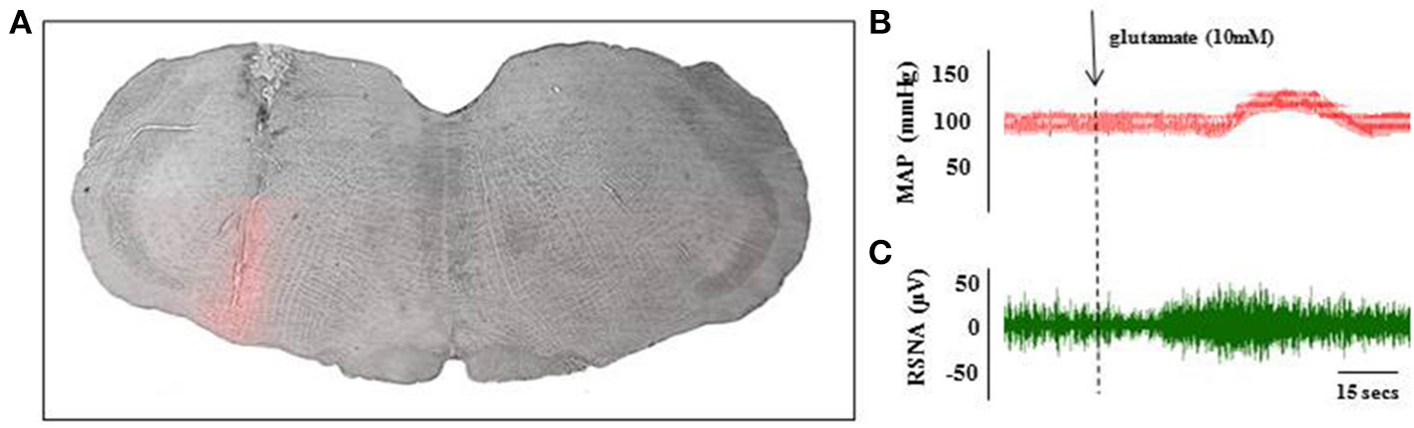
**Histological and physiological verification of RVLM injection site**. A subset of animals received Chicago Blue injection at the end of the glutamate identification procedure to verify histological placement of pipette. Coronal sections through the hindbrain were used to identify the injection site at the level of the RVLM **(A)**. Scale bar = 500 microns. Microinjection of glutamate (10 mM; 40 nL) into the RVLM evoked transient increase in MAP **(B)** and renal sympathetic nerve activity **(C)** within 30 s of injection. These parameters returned to baseline levels within 15 min of the injection.

RVLM neurons were identified by nano-injection of glutamate which elicited a minimum increase of 15 mmHg in blood pressure within 15 s (Figure 3) and an increase in RSNA which preceded the change in blood pressure (Figures 3B,C). The effect of glutamate on blood pressure and RSNA had a short duration; all parameters returned to baseline within 15 min. At the conclusion of the experiments, a subset of animals was injected with Chicago Blue to further verify the location of the injection site (Figure 3A).

#### Microinjection of leptin into the RVLM produced an increase in mean arterial pressure (MAP) and renal sympathetic nerve activity (RSNA)

**Figure 4:**
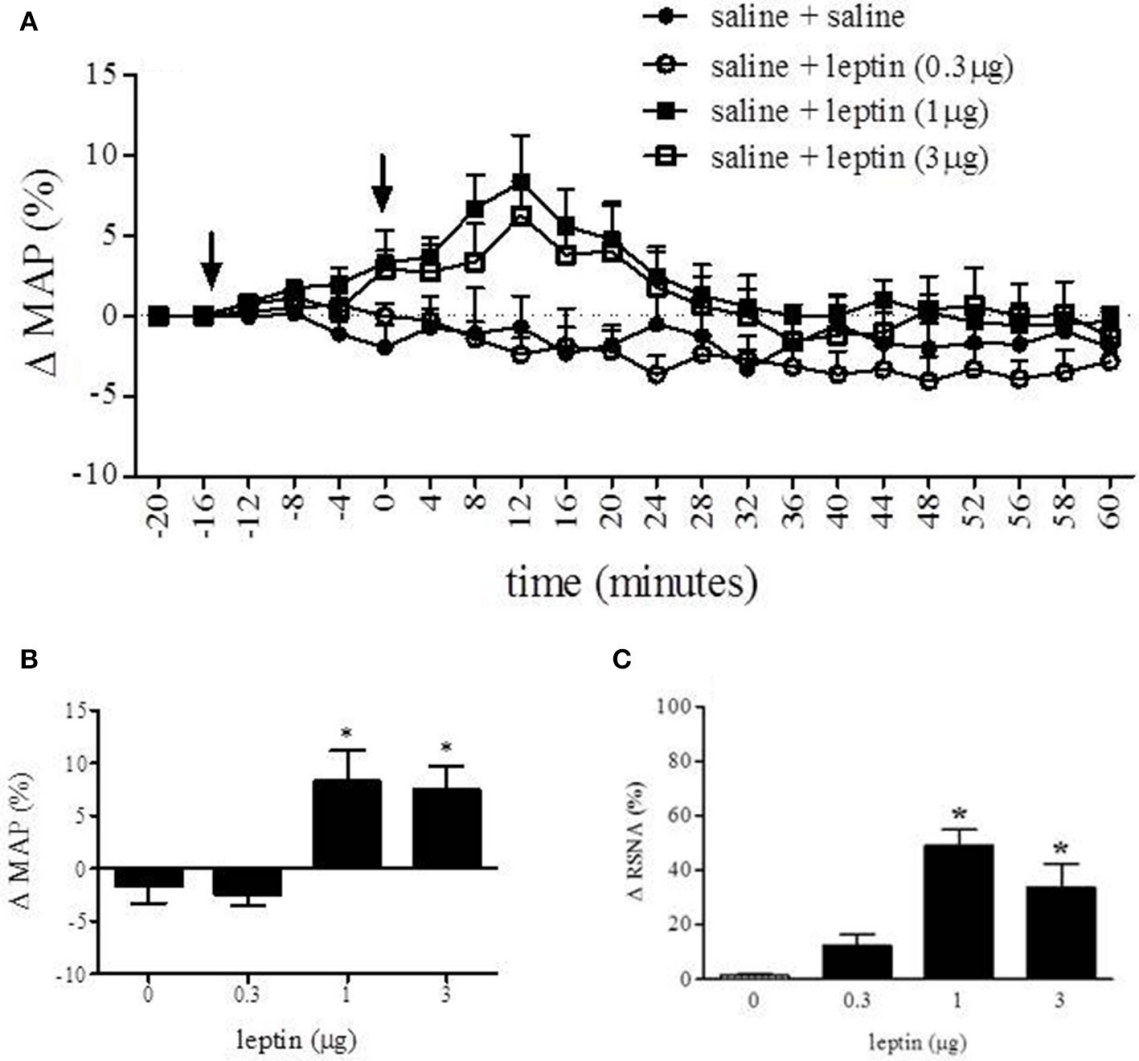
**Nano-injection of leptin into the RVLM increased MAP and RSNA**. Nano-injection of leptin (1 and 3 μg; 40 nL) into the RVLM increased MAP **(A)**. Analysis of percent change in MAP was conducted with One-Way ANOVA which compared the MAP at 12 min following injection between each experimental condition **(B)**. Peak response of leptin (1 and 3 μg) was 8.9 ± 2.9% and 7.6 ± 2.1% respectively. The MAP response to these treatments was significantly different from saline (−1.6 ± 1.6%) and leptin (0.3 μg) (−2.4 ± 1.0%). Leptin (1 and 3 μg) increased RSNA **(C)** 49 and 34%, respectively. (Bonferroni *t*-tests; ^*^p < 0.05); arrows indicate time of first (−15 min) and second (0 min) injection. Analysis of percent change following the first inject was not statistically different from values obtained prior to the first injection.

Long Evan rats received one of three doses of leptin into the RVLM to observe the effect on MAP and RSNA (Figure 4). Figure 4A displays the percent change in MAP over time in response to saline, 0.3, 1, or 3 μg of leptin injected into the RVLM. Analysis of percent change in MAP was conducted with one-way ANOVA which compared the MAP at 12 min following injection between each experimental condition (Figure 4B). In response to leptin (1 and 3 μg) MAP increased 8.3 ± 2.9% and 7.6 ± 2.1% respectively. The MAP response to these treatments was significantly different from saline (−1.7 ± 1.6%) and leptin (0.3 μg) (−2.4 ± 1.0%) [*F*_(3, 14)_ = 8.019; *p* < 0.05]. One-way analysis of MAP prior to and following the first injection (saline) was not statistically different (data not shown).

A One-Way ANOVA was also conducted to compare the effect of each treatment on the percent change of RSNA. There was a significant effect [*F*_(3, 24)_ = 7.493; *p* < 0.05] of leptin (1 and 3 μg) on the percent change of RSNA when compared to saline and leptin (0.3 μg). Leptin (1 and 3 μg) increased RSNA 49 and 34% respectively, whereas saline changed RSNA 1% and leptin (0.3 μg) caused a 12% increase.

#### Microinjection of rat superactive leptin antagonist (SLAN-4) eliminates leptin-induced increases in MAP and RSNA

**Figure 5:**
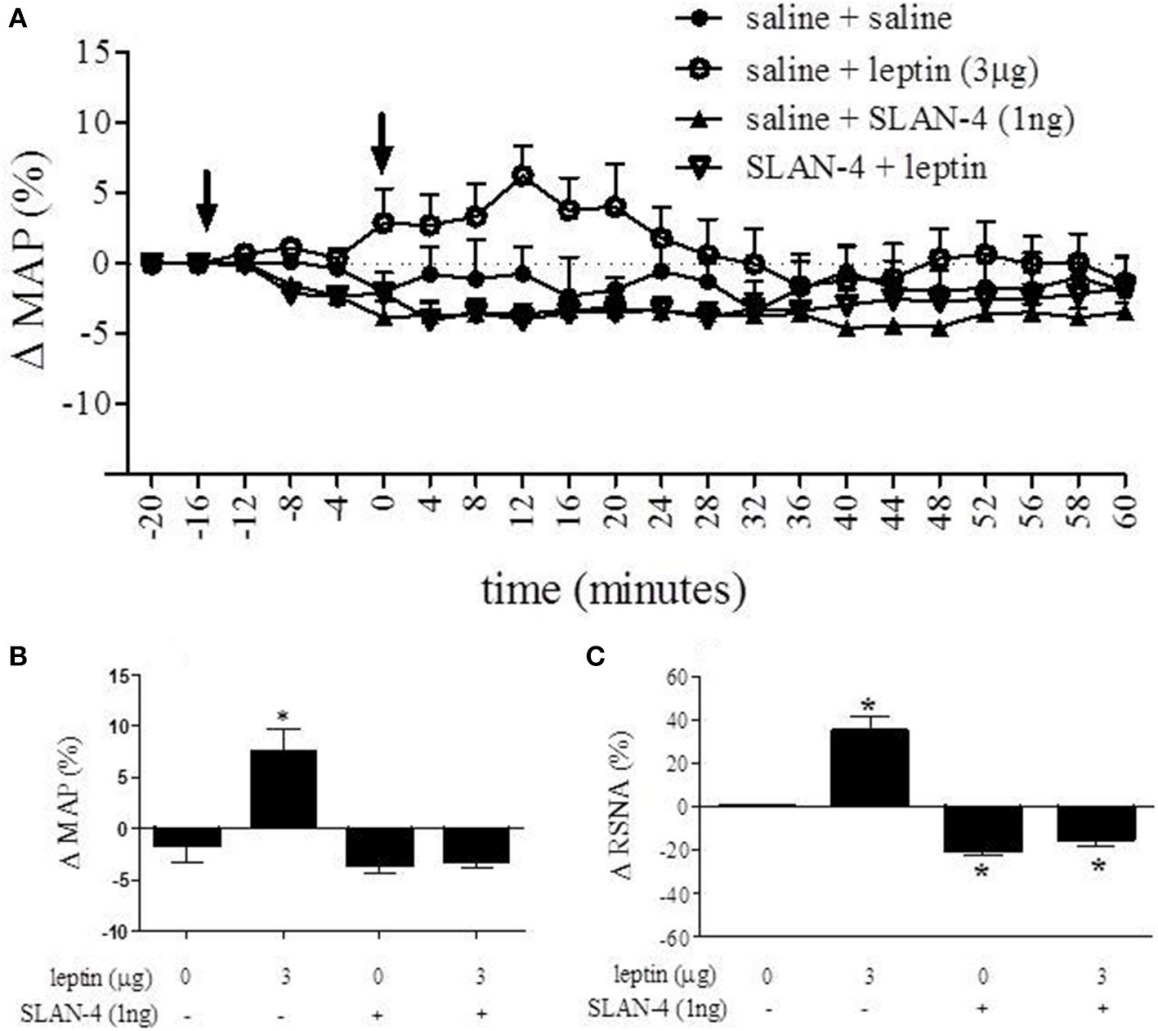
**Nano-injection of rat superactive leptin antagonist (SLAN-4) attenuated the leptin-induced increase in MAP and RSNA and decreased basal RSNA**. Time course response of MAP after administering leptin and SLAN-4 into the RVLM is presented in **(A)**. 12 min following the second injection, MAP was increased following saline + leptin relative to saline + saline controls, while saline + SLAN-4 and SLAN-4 + leptin treatment did not significantly alter MAP relative to saline + saline treatment **(B)**. In contrast, saline + leptin treatment caused a significant increase in RNSA, while saline + SLAN-4 and SLAN-4 + leptin treatment caused a significant decrease in RNSA relative to saline + saline controls. **(C)** (Bonferroni *t*-tests; ^*^p < 0.05).

The role of leptin receptors within the RVLM as it relates to cardiovascular parameters was assessed using the rat superactive leptin antagonist, SLAN-4. The time course response of MAP after administering leptin and SLAN-4 into the RVLM is presented in Figure 5A. A One-Way ANOVA of peak responses measured at 12 min following the second injection demonstrated a statistically significant effect of treatment [*F*_(3, 16)_ = 16.64; *p* < 0.05]. Leptin (3 μg) administration resulted in a 7.6 ± 2.1% increase in MAP while SLAN-4 alone resulted in a 3.6 ± 0.8% decrease at the same time point. When SLAN-4 was administered into the RVLM prior to leptin (3 μg), leptin administration failed to produce an increase in MAP, and a decrease of 3.2 ± 0.6% was observed. 12 min following the second injection, the MAP responses to leptin (3 μg) alone was significantly different from the response to saline, SLAN-4, and SLAN-4 plus leptin (3 μg). The MAP response to SLAN-4 alone and SLAN-4 plus leptin (3 μg) were not significantly different from saline (Bonferroni *post-hoc t*-tests; *p* < 0.05) (Figure 5B). A One-Way ANOVA of percent change in RNSA across treatment was statistically significant [*F*_(3, 19)_ = 8.288; *p* < 0.05]. Following saline injection, leptin (3 μg) administration resulted in a 34% increase in RNSA while SLAN-4 resulted in a 20% decrease. When SLAN-4 was administered into the RVLM prior to leptin (3 μg), a 13% decrease in RNSA was observed. The change in RNSA in response to leptin (3 μg), SLAN-4, and SLAN-4 plus leptin (3 μg), were all significantly different from saline control injections (Bonferroni *post-hoc t*-tests; *p* < 0.05) (Figure 5C).

## Discussion

Our previous work demonstrated the presence of leptin receptor (ObRb) staining on tyrosine hydroxylase positive neurons in the medulla which make up the C1/A1 cell group (Barnes et al., 2010). The current report has built on this observation and further quantified the relative distribution of leptin receptor positive (ObRb^+^) cells in various nuclei of the ventral medulla. We have demonstrated that the vast majority of ObRb^+^ cells are present in C1/A1 cell column and this staining extends along the complete anterior to posterior extent of this cell group (Figure 1; Table 1). Several ObRb^+^ cells were also observed in the caudal raphe, but the relative number of cells present was minor in comparison to C1/A1 cell group. Furthermore, through the use of PRV, a polysynaptic retrograde tracer, we demonstrated that a substantial subpopulation of neurons that express leptin receptors (ObRb) projects to the kidney. We determined that a large percentage of kidney projecting neurons in both the C1/A1 cell group and the A5 cell group express ObRb, approximately 67 and 85% respectively. We quantified additional neurons that had leptin receptors and projected to the kidney in the VMM and caudal raphe, approximately 45 and 22% respectively (Table 1).

As stated previously, the labeling pattern resulting from our renal PRV injections is consistent with a number of previous studies utilizing the same or similar techniques (Strack et al., 1989a; Schramm et al., 1993; Huang and Weiss, 1999; Sly et al., 1999; Weiss et al., 2001). Injections of trans-neuronal tract tracing into the cortex of the kidney have been consistently shown to retrogradely label hindbrain neurons within the RVLM, VMM, A5 cell group, and the caudal raphe. However, our observation that significant subsets of these labeled neurons are ObRb^+^ is completely novel. Previous studies investigating the phenotype of kidney projecting hindbrain neurons have reported that a subpopulation of these cells express tyrosine hydroxylase (TH), phenylethanolamine-N-methyltransferase (PNMT) and/or 5-hydroxytryptamine (5-HT), i.e., are catecholaminergic, noradrenergic, or serotonergic neurons (Huang and Weiss, 1999). Additionally, it has been shown that a small subset (<15%) of neurons in the ventral hindbrain that projects to the kidney express nitric oxide synthase (nNOS). Further studies will be needed to identify which of these neuronal subpopulations also express ObRb.

Microinjection of leptin into the RVLM, the rostral most subdivision of the C1/A1 which houses neurons associated with control of vasomotor tone and blood pressure (Pilowsky and Goodchild, 2002; Guyenet, 2006), caused an increase in both MAP, approximately 9 mmHg, and RSNA, approximately 50%. Furthermore, when microinjection of leptin (3 μg) was preceded by focal administration of the leptin antagonist, SLAN-4 (1 ng), this leptin mediated cardiovascular response was eliminated. In addition, microinjection of SLAN-4 into the RVLM, with or without leptin co-administration, caused a significant reduction in RSNA, approximately 13 and 20% respectively. The response produced by the highly specific leptin antagonist, SLAN-4, alone suggests that leptin receptor activation in the RVLM has an effect on tonic RSNA activity. These data are the first demonstration that neurons within the RVLM express functional leptin receptors and can respond to leptin by increasing RSNA and mean arterial blood pressure. Taken together, our results suggest that leptin may regulate renal sympathetic tone and ultimately blood pressure by modulating the activity of neurons within the RVLM.

Leptin's influence on cardiovascular dynamics is well established. Intracerebroventricular (ICV) injection of leptin has been shown to increase RSNA and/or MAP in a variety of animal models (Dunbar et al., 1997; Casto et al., 1998; Matsumura et al., 2000; Rahmouni et al., 2003; Rahmouni and Morgan, 2007; Prior et al., 2010). Furthermore, direct microinjection of leptin into discrete hypothalamic nuclei (i.e., ventromedial, dorsomedial, arcuate, and paraventricular nuclei) and the nucleus of the solitary tract (NTS), leads to increases in MAP and RSNA (Marsh et al., 2003; Shih et al., 2003; Montanaro et al., 2005; Rahmouni and Morgan, 2007; Mark et al., 2009; Ciriello and Moreau, 2012). In the majority of these studies, changes in both MAP and RSNA were observed with peak changes observed at approximately 15–25 min post injection. These response dynamics were similar to those observed in the present study (see Figures 4A, 5A). The magnitude of our observed leptin induced changes in MAP were also similar to the above microinjection studies which reported changes in MAP from approximately 8 (Marsh et al., 2003) to 17 mmHg (Shih et al., 2003; Rahmouni and Morgan, 2007), with the exception of Ciriello and Moreau (2012), which reported a maximum increase of approximately 32 mmHg following microinjection into the NTS. In contrast, our leptin mediated increases in RSNA were slightly less than that reported in other microinjection studies, which ranged from approximately 65% (Ciriello and Moreau, 2012) to 110% (Mark et al., 2009). Furthermore, the dose of leptin required for maximal response in both MAP and RSNA in the current study (1 μg) was similar to that reported by Mark et al. (2009) in the NTS, but greater than that required by Ciriello and Moreau (2012) in the NTS (0.1 μg) and other studies utilizing microinjection of leptin (0.02–0.5 μg) in hypothalamic nuclei (Marsh et al., 2003; Shih et al., 2003; Rahmouni and Morgan, 2007). These differences may be caused by the reduced density of ObRb receptors in the RVLM (see Figure 1) relative to NTS (e.g., Barnes et al., 2010) and the hypothalamus (e.g., Zhang et al., 2011).

One of the most intriguing finding in the present study is that pharmacological blockade of leptin receptor signaling in the RVLM via the newly developed leptin antagonist, SLAN-4, lead to decreased MAP and RSNA. SLAN-4 is a rat superactive leptin antagonist with similar properties to the recently developed mouse superactive leptin antagonist (Shpilman et al., 2011). These mutated leptin molecules have been shown to have potent effects on feeding and body weight *in vivo* (Elinav et al., 2009; Shpilman et al., 2011; Chapnik et al., 2013). These data provides further support to the role of leptin in regulating cardiovascular parameters. Indeed, administering leptin results in a significant increase in blood pressure and renal sympathetic activity. Taken together, our findings suggest that endogenous leptin may act locally at the level of the RVLM to influence blood pressure in the normotensive state.

## Author contributions

Maria J. Barnes and David H. McDougal were involved in the study concept and design, performing the experiments, acquisition, analysis and interpretation of data, and drafting of the manuscript.

## Grant support

Research reported in this publication was supported by: The National Institute of Diabetes and Digestive and Kidney Diseases under Award Number K01DK078588, the National Institute of Neurological Disorders and Stroke under Award Number NS55866 and National Institute of General Medical Sciences under Award Number GM103528 of the National Institutes of Health. This work used the facilities of the Pennington Cell Biology and Bioimaging Core, which is supported in part by COBRE (NIH 8 P20 GM103528) and NORC (NIH 2P30DK072476) center grants from the National Institutes of Health.

### Conflict of interest statement

The authors declare that the research was conducted in the absence of any commercial or financial relationships that could be construed as a potential conflict of interest.
